# Supplementary material for: Designed for simplicity, used for complexity: The systemic pressures shaping walk-in clinic practices and outcomes
Source: PLoS One. 2025 Jun 9;20(6):e0325793. doi: 10.1371/journal.pone.0325793 (PMC12148145; doi:10.1371/journal.pone.0325793)
Supplement: S1 File — (DOCX) [file pone.0325793.s001.docx]

**S1 – Interview Guide**

Manuscript ID: PONE-D-24-57353

Manuscript Title: Designed for simplicity, used for complexity: The systemic pressures shaping walk-in clinic practices and outcomes

***Preamble***

Thank you for agreeing to participate in this interview. The purpose of this interview is to explore your experiences providing walk-in care, perceptions around variations in clinical decision-making between walk-in care and comprehensive primary care, and your suggestions on strategies to facilitate quality improvement. The interview should take approximately 45-60 minutes. If any question makes you uncomfortable, you can skip that question. You may stop participating at any time. With your permission, I would like to audio record the interview because I don’t want to miss any of your comments. May I turn on the audio recorder?

1. Can you describe your experience providing walk-in care in Ontario?

Probe: length of experience, number & type of practice settings, patient population, healthcare needs, mode of service (physical/virtual walk-in care), workload

At follow-up:

1. [If participant has worked in multiple clinics] Has this differed across settings?
2. [If participant has experience in comprehensive primary care] How does this differ from traditional primary care?
3. Are there differences in the type of care provided for physical or (and) virtual walk-in care compared to comprehensive primary care?
4. What factors prompted you to work in a walk-in clinic setting/providing physical or (and) virtual walk-in care?

Probe: remuneration, organization, scheduling, patient population

1. What system-level factors influence the clinical decision-making when you provide physical or (and) virtual walk-in care?

Probe: policies and procedures, infrastructure, system resources

1. What clinic-level factors influence your clinical decision-making when you provide physical or (and) virtual walk-in care?

Probe: resources, patients, colleagues

1. What physician-specific factors may influence your clinical decision-making when you provide physical or (and) virtual walk-in care?

Probe: knowledge, attitudes and beliefs about the available evidence, confidence in providing care, relationships with other team members, etc.

1. We found a significant difference in short-term healthcare utilization [Indicator 1] between patients who receive physical or (and) virtual walk-in care and those who receive comprehensive primary care. What do you think contributes to this variation?
2. We found a significant difference in short-term healthcare utilization [Indicator 2] between patients who receive physical or (and) virtual walk-in care and those who receive comprehensive primary care. What do you think contributes to this variation?
3. We found a significant difference in short-term healthcare utilization [Indicator 3] between patients who receive physical or (and) virtual walk-in care and those who receive comprehensive primary care. What do you think contributes to this variation?
4. What strategies do you think would help address [insert factors mentioned]?

Probe: regulations, restrictions, incentives, standards

At follow-up:

How would you envision [insert strategy] would be implemented?

1. Is there anything else you would like to mention we haven’t discussed today?
2. As you may recall from the study information letter, we would like to invite you to a follow-up interview to explore factors that influence specific clinical outcomes in the walk-in setting. To achieve this, we will pull outcome data for the patients you have seen in your walk-in practice and discuss your thoughts on how to better support you and your colleagues in providing walk-in care. If you are interested participating in this follow-up interview, I’ll send you the follow-up information and consent form.

Those are all the questions I have for you. I appreciate the time and insights that you’ve given me today.

### **Follow-up Interview (Stage 2)**

***Preamble***

Thank you for agreeing to participate in this interview. The purpose of this interview is to explore factors influencing the walk-in care you provide and the corresponding quality improvement strategies. The interview should take approximately 30-45 minutes. If any question makes you uncomfortable, you can skip that question. You may stop participating at any time. With your permission, I would like to audio record the interview because I don’t want to miss any of your comments. May I turn on the audio recorder?

1. As you look at the data specific to the patients you’ve treated through walk-in clinics, what do you notice? Please think out loud as you review.

Our population-level study found differences in patient outcomes in comprehensive primary care compared to walk-in care. Those differences existed in indicators 1, 2, 3 [to be informed by broader study].

1. Your data for [indicator 1] shows ABC while the average for comprehensive primary care is XYZ. What do you think contributes to this difference?

Probe: individual-, clinic-, system-level factors

At follow-up:

1. What strategies do you think would help address [insert factors mentioned]?
2. How would you envision [insert strategy] would be implemented?
3. Your data for [indicator 2] shows ABC while the average for comprehensive primary care is XYZ. What do you think contributes to this difference?

Probe: individual-, clinic-, system-level factors

At follow-up:

1. What strategies do you think would help address [insert factors mentioned]?
2. How would you envision [insert strategy] would be implemented?
3. Your data for [indicator 3] shows ABC while the average for comprehensive primary care is XYZ. What do you think contributes to this difference?

Probe: individual-, clinic-, system-level factors

At follow-up:

1. What strategies do you think would help address [insert factors mentioned]?
2. How would you envision [insert strategy] would be implemented?
3. What are your reflections as we walk through your data?
4. Is there anything else you would like to mention we haven’t discussed today?

Those are all the questions I have for you. I appreciate the time and insights that you’ve given me today.
