# Supplementary material for: Designed for simplicity, used for complexity: The systemic pressures shaping walk-in clinic practices and outcomes
Source: PLoS One. 2025 Jun 9;20(6):e0325793. doi: 10.1371/journal.pone.0325793 (PMC12148145; doi:10.1371/journal.pone.0325793)
Supplement: S2 File — (DOCX) [file pone.0325793.s002.docx]

**S2 – Supporting Quotes**

Manuscript ID: PONE-D-24-57353

Manuscript Title: Designed for simplicity, used for complexity: The systemic pressures shaping walk-in clinic practices and outcomes

**Theme I:** **Current system demands create strain within the WIC model**

**Repeat Visits**

*“Perhaps patients are coming in for chronic issues, right. So, I think they may see the walk-in clinic doctor to get a prescription for their blood pressure medication, for which they have not been able to see their family doctor. It looks like they're seeing the walk-in clinic doctor and the family doctor for the same problem at the same time, but essentially, it's a chronic problem, for which they keep on needing to see someone.*

*People see walk-in doctors for, I don't know, shoulder pain, shoulder injuries, for example. Again, so you know, the walk-in doctor may order some tests, and then tell them to see the family doctor for follow up. […] So, I think most problems that you see in the walk-in clinic need more than one appointment. The second appointment is often to follow up on results. So, I think that's the reason why the numbers seem that perhaps patients are double doctoring. But that's not really true. I think.” P1*

*“I think it really does depend on the clinical scenario. […] So, you know, if you have complicated medical issues and multiple comorbidities and you’ve come to a walk-in clinic physician for advice in the moment and they’re making medication changes without the broader context of the whole history, yeah.*

*I think in that scenario, it does make sense to tell the patient to follow up with their family physician to let them know that I changed this person’s medications, right? You know, in the context of what might be years of endocrinology notes, does it make sense? So, I don't think in that scenario.” P10*

*“I would also add that as walk-in doctors because we don’t know these patients, there’s a lack of trust on our end too. There are so many patients who are like, I’m taking a past medical history and they’re like, “No, healthy,” and then you start asking questions and they’re like, “Well, actually because of my thyroid issue,” and you’re like you just said that you were healthy otherwise. So, I think the fact that again the time constraint and just not knowing the patients, I think a lot of times walk-in doctors probably err more on the side of caution. It’s just fail-safe advice to protect ourselves and be like, “Well, if you’re not getting better, then follow-up.” P18*

*“I've had patients come and say, well, I was at the walk-in this morning, and now I need to talk to you about the same issue in the same day even. And then if you try to clarify, well, no, doesn't really work that way, patients get very upset. It's all education. I've had patients say to me, I went to the walk-in yesterday, and I just wanted to let you know, I wanted to just tell you that I got treated for this. And that's a whole visit. They don't know that you don't need to book a visit with me […].*

*And I think it's also just if one treatment is given, and it's not working right away. That's a common reason. I see people too, like I was treated yesterday and I'm not immediately better. Again, that's communication and education of maybe just not knowing that's not what we would have expected.” P3*

*“The other part is as well the expectations for turn around on part of patients. So, often times I will say to somebody you know give this medication 48 hours to work. If you’re not any better two days from now, follow up. And they’ll follow up the next morning. Or I’ll say you know I recommend a few weeks of physio. Follow up if you’re not improving as expected by this number of weeks and they’ll come in next week because they had two physio sessions and they’re not better.” P4*

*“It’s hard to say, because sometimes a lot of it can be due to patient factors. So, patient factors like, I think there’s just increased patient anxiety, or you know, sometimes patients can go on the internet, and they see something, and then it makes them anxious, and they want to follow up again about it, right.” P9*

*“Some patients are just very anxious. I have this lady with a cold who saw me five times in two weeks, which drove me crazy. She also saw a walk-in doctor at another clinic. So that's six times. No amount of doctor visits can reassure her. So definitely a combination of factors.” P14*

*“I think it’s also the accessibility, right. Sometimes that can be a factor, like I mentioned, there’s so many telemedicine services; a patient could potentially book with all of them, just so that they can be seen now.*

*So, there is that kind of higher demand factor, immediacy of care. Right now, people have higher expectations, right. For example, you know, why would I wait two hours when I can see someone in ten minutes? Why, when I order a package from Amazon, why would I wait two weeks to get it when I could get it the next day?*

*So, a lot of those kind of societal factors are driving patient anxiety and demand to get faster care, I guess. And I think that’s what results in people booking several visits, because in case I didn’t get this one, I can always get that one.” P9*

*“Yeah, absolutely, for sure. That’s, I think, multifactorial. First, patients demand to be seen when they want to be seen. And so, if you call your primary care provider, your primary care provider says, “I could see you in three days.” But you believe your health care issue is more pressing than that. You’re going to go where you need to be seen, right? So, you might book an appointment in three days, but you’re still going to see a walk-in doctor who will temporize your health care condition.” P10*

*“But I do think, you know, certainly having watched – you know, I’ve practiced in the last 15 years in the system. I mean, I think patients have certainly moved to a little bit more of a demand system, right? When they want to be seen, they do want to be seen. And the fact that there are more options available to them means that they will take advantage of them. So, if you don’t – if you’re in primary care – and don’t see your patient within the time that they feel is reasonable. Some patients will seek care in a walk-in. Some will seek care in a virtual urgent care program. Some will go to the emergency department. And I think that that’s – it doesn’t mean that they won’t follow up with you, but it does mean that they will seek the care when they want.” P10*

*“When [a patient] sees a walk-in doctor, most of the times it’s because they want to be seen immediately. So many times, they’ll come in and say, “Oh I have an appointment with my doctor in two days, but I didn’t want to wait two days”. So, they see us first and then they get a second opinion from the family doctor two days later, because that’s when the family doctor is available. And that two days might be a very appropriate triaging of that issue. It’s probably not urgent and two days is probably reasonable. But from a patient perspective, if they’re coughing or they have an immediate symptom, they feel that it needs to be addressed immediately. So again, it’s that expectation that the family doctor should be available immediately.” P11*

**Potentially Unnecessary Antibiotic Prescriptions**

*“It’s the self-selection of patients to some degree. Someone’s prescribed me antibiotics in the past. Oh, I went to this clinic, and they did. I’ll go back to that clinic, and I’ll get the same thing.” P5*

*“It’s just the demand for care. Like, walk-in clinics are going to see usually 75 to 100 percent more patients a day than a family doctor. So, you want to see those patients, you feel stressed because they’re waiting a long time, and the more that they wait, the more demanding that they will become. So, you want to be very efficient. And so, sometimes you realize it’s not worth arguing with the patient and practicing good medicine. You might as well just give them the prescription so that you can move on to the next patient.*

*So, there’s stress and pressure placed on us just because of the volume that we have to see. We have to see that volume. They’re waiting. They’re getting angry. You want to also leave at a reasonable hour so you can see your family. So, you don’t want to spend a lot of time. And sometimes, like, did you get anything out of 10 minutes out of arguing? You might just order the script anyways and that’s another patient you could have seen.” P11*

**Theme II:** **Efforts to address system demands expose misalignments within the WIC model**

**Repeat Visits**

*“[Repeat visits are] extremely common for several reasons. The first one being that likely the walk-in physician has advised the patient to follow up. And for multiple reasons, they often do it to cover themselves medico-legally and to make sure that they don't get lost to follow up, or like slip through the cracks. And then also, because ultimately, they're often issues that need follow up regardless, or if they've ordered tests to make sure that they follow up with the family doctor, because if they have access to someone that has continuity of care in their circle, then they might as well continue with that person.” P2*

*“That can be for several reasons. One is like they’re often told to follow up with their family doctor. This is standard advice because most good doctors recognize that they might not get it right the first time and need to get followed up. So, that would be the most benign reason in my mind.” P6*

*“I think it’s fine if they come back and follow up with us. It’s just that you don’t know the patient well enough that, just in case something was missed during the encounter, some sort of medical condition you had no clue about, “Just in case if things get worse see someone again because I don’t really know you really as a patient, right?” So, it’s always erring on the side of caution in that sense, whereas a family doctor has all of their medical history and also knows them as people. […] They know these patients and they can give the proper advice, and we can’t do that. [laughs] So we’re always going to err on the side of caution.” P18*

*“They could perhaps feel that way because of the quickness of the visit, the demeanor of the physician, sometimes even something as simple as the tone of the physician. And they didn't get what they want, like the patient didn't get what they want. And they think that because patient satisfaction wasn't optimized that patient care isn't good enough. And that's not, they don't equate to one another.” P2*

*“You know what I would say A you’re spot on that this is a problem. Either the family physician is a second opinion after they’ve gone to the walk-in clinic or vice versa.*

*I personally don’t encourage it, but the unfortunate fact is Ontario physicians are more terrified than ever about making patients angry or setting realistic expectations simply because the College did not support us when we tried to explain how to best navigate the system despite limited resources. Patients do not want to hear that.*

*What they want to hear is that they can have their burger their way like it’s a McDonalds. They can access care here; they can access there. And they can go to the family physician because they want reassurance that this other doctor, they don’t know, is doing the right thing or making the right recommendations. Sometimes they follow up because disappointingly the walk-in physician did not do a thorough job. They didn’t assess them thoroughly. They didn’t order any tests, and the patient really thought that something needed to be done.” P4*

*“Well, I think walk-ins can be really busy, so sometimes patients will feel that their issue is not being properly addressed. So sometimes physicians can be very rushed and busy and not empathetic. So, there’s definitely more of that, I think, in walk-ins than primary care. I think with primary care because you’re incentivized to take your time and to keep people healthy and different payment models; you have a much better relationship with patients. Whereas the walk-in doctor will just kind of do the basic necessities and suggest that they follow up with their family doctor for the deeper issues.” P11*

*“And maybe I put my emphasis on the patient’s expectations and worries in walk-in because I think more people when they go to a walk-in there’s a bigger expectation of this is what I am looking for.*

*And a lot of the time I’d say what they’re looking for isn’t the correct option. And maybe it’s because they’ve gone to their family doctor asking for this certain thing and the family doctor has said no, that they’re trying somewhere else to see if they can get it from there. And so, I probably say no more at a walk-in than I would in a family practice.” P5*

**Potentially Unnecessary Antibiotic Prescriptions**

*“So, I think even despite optimal communication sometimes just the expectations are that they will always have someone they can just pop in and talk to for reassurance or to express their frustration that they’re not getting better as fast as they want to.*

*They’ll just come in every day for three or four days in a row to the walk-in clinic just to make sure they don’t need antibiotics because you know there’s an expectation that there should be antibiotics for a viral illness. But that, there may be a cultural thing. I find in some countries you can get antibiotics for UTI’s. You get Amoxicillin over the counter. And so those expectations that you can just walk in and get something or, is kind of ingrained in them. So, now the only step that’s in the way is the physician.*

*So, they just walk in, they talk to a physician and say oh, can I have some antibiotics. And oh, if I’m not better tomorrow can I get some antibiotics tomorrow? I think that’s another part of the, the repeat visits that are not necessary.” P4*

*“You’re just pressured by financial factors, by your management, by patients, by basically everybody telling you to just give them what they want and get them out the door.” P6*

*“You can try to explain things. But the patient may not understand. They might ask more questions. And now you have a more time-consuming appointment, which decreases your pay. Overall, it's not worth it. It's too much effort. It's less pay.” P14*

*“In a walk-in clinic, there’s the issue of volume, because you have to get paid, because you only get paid for the number of patients you see. Taking the time to convince a patient that they don’t need the thing that they came here for the purposes of getting, that’s a lot of – you know, it takes a certain amount of energy.” P16*

*“Honestly, it’s the payment model, right? I mean if I’m salaried, I’ll take my time talking to you and counselling you about why you shouldn’t be taking antibiotics for this cold, but if I am on a fee-for-service model, what’s the point in giving you counselling for 15 minutes, which is not paying me, right? I’d rather see three more patients in that window for that compensation.” P17*

*“I think because time is so precious and so minimal in those settings, that it's just easier to satisfy the patient. And because it's faster just to move on and go to the next patient. It's not in the clinic's best interest for the walk-in clinic doctor to sit and have a 10-minute conversation with the patient about why they don't need an antibiotic.” P2*

*“[Walk-in clinics] should not just be antibiotic prescription factories, which is what I feel like a lot of them are. They need to practice proper medicine but unfortunately, I feel like the model attracts the worst doctors.*

*It seems like the ones that are the least skilled and the least up to date, because no one looks at their practice. They can make people happy by doing the wrong thing like handing them an Amoxicillin prescription when really, they just need to be told to go home and wait. Yes, unfortunately it attracts those kinds of practitioners.” P6*

*“The people are yelling at you and like swearing at you and it’s like the tenth person who’s yelling and swearing at you in that shift. Not even that day. Just like for this four-hour shift there’s another person yelling and swearing at you and you’re just trying to do your job properly.” P6*

*“Plus, with the volume most walk-in physicians see, I think there’s compassion fatigue from the clinicians. And so, both sides create a circumstance that’s more likely to be cognitively and emotionally straining for both parties. And in lieu of the difficult conversation and perhaps the burnout with just trying to provide basic scientific education, it may be easier to provide quote/unquote an unnecessary medication.” P19*

*“So, I think physicians feel pressured to give patients what they want because there is an element of customer service in medicine these days, unfortunately, right? So, I think it's almost like the customer is right. Otherwise, they're going to leave you a one-star review on rate MD, right. So, I think that kind of pressure puts physicians in a tight spot to do unnecessary treatments for patients’ satisfaction, which is now a big deal. And patient satisfaction and following the guidelines, etc., are not completely in sync.” P1*

*“You know, I think it can happen in family practice, but when you have a good enough relationship with your patients in family practice you can educate them. They trust you. So, I'll often have most of my patients saying, “Well do you know what? If you think it doesn't need it.” And I'll give them the follow-up. “If you're not better in two days come back and we'll talk about other options.” So, there's that continuity of care and that follow-up, which gives them the comfort. Again, when you manage the education side of it, you're able to manage the outcomes a little bit.” P15*

*“Say for example, a patient comes to you and says I'm having certain pain symptoms that are typical of a bladder infection. You as a walk-in physician have to take their word for it, because you don’t know them, you don’t know their history, you don’t know that their typical bladder infection looks like, and you are more likely to treat that.*

*But if you are their family physician, you might look back and say, “Hey, this is your sixth episode this year; why don’t we get a urine sample first; let’s try and wait for the results, see how you're doing in two days.” You know?” P16*

*“Again, they do that to expedite [the visit]. I think the patient history matters a lot too. Not knowing about the past medical history. If a patient comes to you and tells you this is what I have and I know this is what it is, you kind of have nothing to go off of but their word, right?” P17*

**Theme III: These misalignments result in trade-offs between cost-efficiency, accessibility, and care quality**

**Repeat Visits**

*“Walk-in clinics are not funded, are not resourced to do some of these more complex procedures. So instead, what they do is they see these patients, diagnose them with what everybody knows them to have – essentially a laceration or some sort of issue requiring repair – and send them to another provider.” P10*

*“I think it depends on the nature of the complaint they come in with. If it has some really big red flags such as a concussion, right, I will say because it has – something called a post‑concussive syndrome, so there are some conditions that evolve over time, right, and that cannot be assessed in the moment when the injury or the incident happens.*

*[…] For those kinds of patients, I would suggest follow‑up, even if it’s not required, just so that they have some kind of accountability, especially with mental health patients. I tell everybody to follow up with their doctor, right.” P17*

*“If they're anxious, regardless of whether the family physician or the walk-in clinic doctor did a thorough job, they're going to go and get another doctor to see them anyways, because they're anxious about it. […] So, you can't really stop a patient from double-dipping for the same issue. Because ultimately, we can't tell the patient what to do.*

*Healthcare doesn't allow us to say, if you've already seen a patient for this issue, you can't see them again. So, the patient's going to basically have all the power to decide, I'm going to go and see my family doctor anyways, sometimes even on the same day, which can be extremely frustrating, because then that means one of the doctors doesn't get paid for their work.” P2*

**Potentially Unnecessary Antibiotic Prescriptions**

*“And I mean, also on the back of their mind, even if [the walk-in physician] insists it’s a condition that doesn’t need antibiotics, in case of that one per cent where you are treated on antibiotics, and if the patient is not happy, then I mean that could lead them to the trouble. So, I guess the other motivation could be to be on the safer side.” P8*

*“Antimicrobial stewardship is important, but stewardship is looked at in a population setting and this goes again to theory versus practice. When you’re the individual covering for your local community, your main priority is that community, and you have to determine, do have bacterial pneumonia as opposed to viral and then what does that mean for your colleagues? And so, I think that’s kind of that separation of theory and practice because at that point the potential of negative outcomes, one being for example refractory, GI indigestion, diarrhea, yeast infection, inappropriate antibiotics versus decompensated bacterial pneumonia.*

*I think a part of it is also the concern about medical legal issues. And so, you’ve got to pick your poison, which is more concerning to you, right, and which the patient will be more likely to make a complaint about? I presume I’d likely, you’d probably know better than me, it probably is likely more if you were to withhold a treatment as opposed to provide an unnecessary one or an ostensibly unnecessary one, so I don’t know.” P19*

*“But for some doctors, it's less effort for them to just give the medication and they pick their battles where they can.” P2*

*“It’s when you're stressed and you want the encounter to end, the patient comes begging for antibiotics, you try to educate but at some point, they keep pushing and you're like, fine. I'll just give you the antibiotic. You don't need it, but you're not going to go away without getting it, so I'll just give it to you. So absolutely it's when you're busy. It's when you're burnt out. It's when the patient is making a big fuss out of it, even though you really think it's not needed, it happens.” P15*

*“I’ve given antibiotics when I know it won’t help. Unfortunately, that's the case during the flu and cold season. We see so many people come with a sore throat, cough, runny nose, which are all clearly viral, but they will not leave, and they cannot be reassured, no matter how much time you spend with them. When I first started, I really tried to explain to each patient the difference between viral and bacterial infections. That the viruses can go away on its own. We don't need any medications. Whereas for bacterial infections we need antibiotics. And at the end the patient will be like, “So are you going to give me antibiotics?” And I feel like I just wasted my time talking to them. They didn't listen and so sometimes it's just easier to prescribe so they're happy, and I know it’s not good; it contributes to antibiotic resistance, however that's just the reality. When patients come in with a strong idea of what they want it's very hard to persuade them.” P14*

*“I saw a family of three children, all under the age of six. They all had upper respiratory illnesses with conjunctivitis and, you know, a mild cough. None of them required antibiotics. The parents were from another country where they hand out antibiotics all the time, and so they had the expectation of getting antibiotics. So, you know, because I was able to see three people at once with the same problem, I was able to take my time, sit down with the family, educate them on, you know, what a bacterial infection is to what a viral infection is.*

*I provided a lot of reassurance to them. Let them know when to come back, when not to come back. And I was able to make mom and dad feel more comfortable in managing this without any medications. And we didn’t have to prescribe antibiotics to three children unnecessarily.” P11*

*“I think it has to do with the pressure to get through patients. Right? And keep patients, I guess, to some extent happy. And so, patients are happier when they feel like they've been listened to, and they've been given something. Right? So, you know, you wait two hours to be seen in a walk-in clinic, you see a provider for five minutes and they send you out with nothing other than advice and counselling, often times people feel like they didn't receive value, right? You leave with that shiny prescription in your hand, you got value for your visit. And it's a little bit of a human factor but it is – it's real. And I think rightly or wrongly it certainly influences practice in those settings.” P10*

*“Patients who get what they want, always leave happy. Patients who don’t get what they want, you either spend the time and they're like, “Oh, you know what, I understand; thank you for doing that,” but then you're not getting paid for that. And patients who don’t get what they want, and you spend the time and they're still not happy, now you're not getting paid, and you have an angry patient.*

*And that doesn’t feel good on any front, so I think the psychology of all that, it’s easier for walk-in clinic doctors to give patients what they want, one, so that time goes faster, and they get paid more for it. And two, because it’s just tough to say no to people regularly and have them be angry at you.” P16*

*“It’s because I don’t know you; I have to trust that your symptoms are as bad as you say they are. I haven't seen you when you’ve been sick other times for me to say, “Hey, this looks like every other sickness you’ve had that’s gone away without antibiotics.” It’s because I'm not going to have a subsequent follow-up relationship with you where I can say, “Listen, I don’t think you need antibiotics; let’s give this twenty-four to forty-eight hour; if I'm wrong and you're not getting better, just tell my secretary, I will send the antibiotics over.*

*I can't do that as a walk-in clinic doctor; they have to come back; you have to make them wait. It’s just the nature of the relationship doesn’t give you the flexibility to say, “I think you're actually OK. Let’s give this twenty-four to forty-eight hour and reassess.” P16*

*“It’s time, they just want the patient out of the office, and they want to move on to the next one. This is the patient's expectation, on average, per appointment or per visit, it takes significantly shorter to throw an antibiotic script at the patient than it does to educate them to help them understand what a viral infection is, what a bacterial infection is, the symptoms of [each]. And then not just that education, but also having the time for the patient to most often pushback to be like, “Well, no, I still want that antibiotic.” So, it just takes a shorter amount of time to say it in better words, shut them up and get them out the door.” P2*
