# Supplementary material for: Designed for simplicity, used for complexity: The systemic pressures shaping walk-in clinic practices and outcomes
Source: PLoS One. 2025 Jun 9;20(6):e0325793. doi: 10.1371/journal.pone.0325793 (PMC12148145; doi:10.1371/journal.pone.0325793)
Supplement: Attachment — Submitted filename: Response to Reviewers.docx [file pone.0325793.s004.docx]

Laura Desveaux, PhD

Institute for Better Health

Trillium Health Partners

2085 Hurontario Street

Mississauga, ON, Canada	L5B 1B8

Manuscript ID:	PONE-D-24-57353

Manuscript Title:	Misaligned mandates: The systemic pressures shaping walk-in clinic practices and outcomes

We would like to thank the Academic Editor, Faten Amer, and the reviewers for their thoughtful and constructive feedback to help improve the clarity and impact of the manuscript. An itemized response for each comment is presented below. Please note that references to page and line numbers correspond to the revised version of the manuscript.

**Editor’s Comments:**

1. **Funding Disclose:**

Thank you for stating the following financial disclosure:

“Funding was received from the Canadian Institutes of Health Research (CIHR) project grant awarded to Lauren Lapointe-Shaw, Noah Ivers and Laura Desveaux (no. 175285).”

Please state what role the funders took in the study. If the funders had no role, please state: "The funders had no role in study design, data collection and analysis, decision to publish, or preparation of the manuscript."

Please include this amended Role of Funder statement in your cover letter; we will change the online submission form on your behalf.

**Author’s Comments:**

We have revised the Cover Letter to include the statement provided:

The funders had no role in study design, data collection and analysis, decision to publish, or preparation of the manuscript.

2. **Data Sharing:**

We note that you have indicated that there are restrictions to data sharing for this study. PLOS only allows data to be available upon request if there are legal or ethical restrictions on sharing data publicly.

Before we proceed with your manuscript, please address the following prompts:

A.) If there are ethical or legal restrictions on sharing a de-identified data set, please explain them in detail (e.g., data contain potentially identifying or sensitive patient information, data are owned by a third-party organization, etc.) and who has imposed them (e.g., a Research Ethics Committee or Institutional Review Board, etc.). Please also provide contact information for a data access committee, ethics committee, or other institutional body to which data requests may be sent.

B.) If there are no restrictions, please upload the minimal anonymized data set necessary to replicate your study findings to a stable, public repository and provide us with the relevant URLs, DOIs, or accession numbers.

You also have the option of uploading the data as Supporting Information files, but we would recommend depositing data directly to a data repository if possible.

We will update your Data Availability statement on your behalf to reflect the information you provide.

**Author’s Comments:**

We thank the editor for raising this concern. Adhering to PLOS ONE's Data Availability Guidelines for Qualitative Data, we have included relevant excerpts directly in the paper. However, due to ethical restrictions regarding participant consent for data release, additional data cannot be made publicly available as the data may contain personally identifiable information. This decision aligns with Trillium Health Partners’ Research Ethics Board. For further inquiries, interested parties may contact Trillium Health Partners’ Research Ethics Board ([THPREB*@*thp*.*ca](mailto:THPREB@thp.ca)).

To provide a more comprehensive view of the data, and in response to reviewer comments, we have added additional quotes as a Supplementary File. All quotes have been de-identified and prepared in line with governing REB requirements for sharing qualitative data.

3. **References:**

Please review your reference list to ensure that it is complete and correct. If you have cited papers that have been retracted, please include the rationale for doing so in the manuscript text, or remove these references and replace them with relevant current references. Any changes to the reference list should be mentioned in the rebuttal letter that accompanies your revised manuscript. If you need to cite a retracted article, indicate the article’s retracted status in the References list and also include a citation and full reference for the retraction notice.

**Author’s Comments:**

We have reviewed the reference list and did not find any errors. If there are specific references that the editor believes require our attention, please let us know.

**Reviewer’s Comments:**

**Reviewer 1:**

1. **Discussion:**

I am interested in having a deeper understanding about whether there were any concentration or trend of themes according to the provider's age, sex, work division between WIC/primary care office, and career stage. Since this population seems to differ from the typical WIC provider, are these respondents more or less confident in telling a patient "no" for inappropriate antibiotics? Are seasoned providers telling patients "no", but newer providers bending to pressure? Or are these providers enthusiastic being just out of school and able to assert themselves where veteran providers are worn down by the system and therefore allow patients to walk over them? The possible response bias is an aspect that I feel you should spend just a little more time on in the report. I know you already have addressed it partially in the limitations, but I would dig into it just a bit more.

**Authors’ Comments:**

Thank you for your encouragement, comments, and feedback. We have revised the Discussion to include a deeper dive into physician characteristics:

“Our findings align with Rodrigues et al.’s systematic review, which identified complacency and fear as key attitudes linked to increased antibiotic prescribing [76]. Additionally, burnout—another theme raised by our participants—has been shown to influence prescribing behavior. Derricks et al. found that physicians experiencing higher levels of burnout were more likely to prescribe opioids and at higher doses for patients with advanced lung cancer [77]. These findings suggest that external pressures, including emotional and cognitive strain, can shape prescribing decisions across different clinical contexts. Investigating whether there are subgroups of physicians for whom the tendency to prescribe is more prevalent could further illuminate how these factors influence practice patterns in high-volume practice settings [78, 79]. Research from the primary care literature indicates that prescribing behaviors do differ based on physician characteristics. For example, a U.S. study found that female general practitioners (GPs) working in the Veterans Administration were less likely to be in the highest prescribing quartile for benzodiazepines, opioids, PPIs, and antibiotics [80]. Moreover, in a systematic review using an international sample, Baillie et al. found that early-career GPs had lower odds of prescribing antibiotics compared to their more experienced peers (OR 0.23–0.67) [81]. These findings suggest that tailored interventions could be valuable in reinforcing physician confidence and providing them with structured strategies to navigate patient expectations [79]. Although no studies have specifically examined the effectiveness of tailored interventions for prescribing decisions, Joosen et al. demonstrated that occupational physicians significantly improved adherence to mental health guidelines after receiving multiple-session peer training designed to identify and address implementation barriers using a Plan-Do-Check-Act approach [82]. Future research could build on this framework by developing physician personas, similar to the work of Shuldiner et al. [83], to inform the co-design of tailored intervention strategies.” (Page 15, Line 6–28)

We also elaborated further on response bias as a limitation:

“These findings reflect the perspectives of Ontario-based physicians trained in family medicine, and may not be representative of physicians from other regions. Unlike recent studies that found WIC physicians are typically men in the late stages of their careers [10], our sample had a larger proportion of women who were early-career. In particular, a study by Lapointe-Shaw et al found that across the 597 WIC physicians practicing in Ontario in 2019, 67% were male with an average years of experience of 25.5 [10]. By contrast, 63% of our participants were female and 47% were early career (vs. 8.5% reported by Lapointe-Shaw et al. [10]). As the intention of this study was to generate insights into what might be contributing to system outcomes, these insights should not be taken to be representative of all family physicians. Future work should explore whether these perspectives resonate with a larger sample of WIC providers, as well as investing whether and how different physician characteristics influence their approach to patient care. For example, more experienced providers may have developed effective strategies to manage patient expectations, while newer providers may be more vulnerable to patient pressure. Conversely, early career physicians may be more familiar with current evidence and firm in their approach to clinical care. Identifying the beliefs that drive physician decision-making and among which physicians these beliefs are most common is a necessary precursor to developing effective strategies to improve population outcomes.” (Page 16, Line 22 – Page 17, Line 10)

2. **Supplemental Materials**:

Finally, I request that you provide additional information in the supplemental data. I would like to see what the interview guide is and also a kind of aggregation of quotes that support the different themes / key messages from across the interviews (i.e., not just the quotes put in the manuscript). This would support that the theme was shared rather than sourced from only one person.

**Authors’ Comments:**

We have added the Discussion Guide and a document containing a broader selection of quotes beyond those featured in the manuscript. These quotes are organized by theme to help readers appreciate the nuance of each theme and the corresponding coverage across participants. We hope these additions address the reviewer’s concerns and further support the validity of our findings.
