# Supplementary material for: Designed for simplicity, used for complexity: The systemic pressures shaping walk-in clinic practices and outcomes
Source: PLoS One. 2025 Jun 9;20(6):e0325793. doi: 10.1371/journal.pone.0325793 (PMC12148145; doi:10.1371/journal.pone.0325793)
Supplement: Attachment — Submitted filename: May 15 - PLOS One - Response to Reviewers.docx [file pone.0325793.s005.docx]

**Response to Reviewers**

We would like to thank the editor for their clarifying questions to improve the manuscript. An itemized response for each comment is presented below.

**Editor Comments**

**1. Sample size: The sample of 19 physicians is relatively small, with the added consideration of the majority of respondents being early career women. As noted in Discussion and Limitations, this is an important consideration.**

**Response:** We have added the following to the limitations section to ensure due consideration:

*“Unlike recent studies that found WIC physicians are typically men in the late stages of their careers [10], our sample was largely composed of early-career women. While our participant sample supported in-depth insights from those actively navigating the landscape in both WICs and comprehensive primary care, future work is needed to understand whether and how these insights resonate with other physician populations, whose perspectives may differ in meaningful ways. Early-career women physicians may experience unique pressures, and future work should explore variability in experiences related to workload, patient expectations, or role flexibility across different physician demographics.”*

**2. Ontario, Canada health service delivery: Additional detail regarding the differences and similarities of service delivery in Ontario, as a Province in Canada, and Walk in Clinics (WIC) and Urgent Care would be helpful. For example, it appears both do not have access to comprehensive medical records. However, WIC may be able to provide dental and eye care, whereas only Urgent Care can treat fractures and injuries requiring stitches? Are the majority of WIC staffed by family physicians or do emergency medicine physicians play a role as well?**

**Response:** We have added the following context at the end of the ‘Setting’ section (note that WICs in Ontario do not provide dental or vision care).

*“WICs and urgent care centres in Ontario operate independent of comprehensive primary care settings and as such, do not have access to comprehensive electronic medical records. Both are often staffed by family physicians, some of whom have emergency medicine training.”*

**3. Renumeration: Is it a national or provincial policy regarding the “penalty” triggered for accessing WIC before primary care, or does that also relate to urgent care?**

**Response:** We have added more context to the Setting subsection as outlined below:

*“An access-related bonus contributes additional income that is reduced when enrolled patients visit WICs in-person or virtually. This disincentive is a provincial policy that applies specifically to visits coded as primary care, such as WIC visits and some urgent care visits.”*

**4. “Misaligned Mandates” is an interesting title – it would be helpful to learn more about the actual legislation or mandates.**

**Response:** The initial title refers to implicit expectations rather than formal mandates. To avoid confusion for those who aren’t familiar with the context, we have updated the title to be more explicit: **Designed for Simplicity, Used for Complexity: The systemic pressures shaping walk-in clinic practices and outcomes.**
